# Long-term water temperature reconstructions from mountain lakes with different catchment and morphometric features

**DOI:** 10.1038/srep02488

**Published:** 2013-08-22

**Authors:** Tomi P. Luoto, Liisa Nevalainen

**Affiliations:** 1 Research Institute for Limnology, University of Innsbruck, Mondseestraβe 9, 5310 Mondsee, Austria; 2 Department of Geosciences and Geography, University of Helsinki, P.O. Box 64, 00014 University of Helsinki, Finland; 3 Department of Environmental Sciences, University of Helsinki, Niemenkatu 73, 15140 Lahti, Finland

## Abstract

Long-term water temperature records are necessary for better understanding climate change impacts on freshwaters. We reconstruct summer water temperatures from three climatically sensitive mountain lakes in Austria using paleolimnological methods aiming to examine long-term thermal dynamics and lakes' responses to regional climate variability since the Little Ice Age. Our results indicate divergent trends for the lakes. In two of the lakes, which are located at the sunny southern slope of mountains, water temperature has increased several degrees concurrent with the observed air temperature increase. In contrast, no change is observed in the reconstructed water temperatures of a shaded lake, located at the northern slope, where also the ecological and thermal changes are most subtle. The results indicate the importance of cold water inputs, such as snowmelt and groundwater, on lakes' thermal conditions and suggest that watershed characteristics and lake stratification play a major role in defining the lake-specific thermal regime.

Climate change causes significant limnoecological alterations in remote freshwater lakes^1^,^2^,^3^, while the patterns in air and water temperatures are likely to be complicated and site-specific^4^,^5^. Since lake responses are often locally forced, there are great challenges in determining consistent long-term trends for different sites. This is an intrinsic problem in paleolimnological sediment studies because environmental forcing factors can be either local or regional^6^,^7^ and they can also vary in time^8^. The present study lakes (Fig. 1) have previously been judged to be climatically ultrasensitive, as Twenger Almsee (2,118 m a.s.l.) and Oberer Landschitzsee (2,076 m a.s.l.) have anomalously warm average summer water temperatures compared to lakes at similar altitude in the region^9^. In contrast, water temperatures in Moaralmsee (1,825 m a.s.l.) are anomalously cold due to slower snowmelt caused by topographic features that reduce sunlight and influence wind impact, and due to its partial location on a moraine field, which makes the basin mostly groundwater fed^9^,^10^,^11^. Moaralmsee, which summertime water temperatures have been documented to be 6–7°C cooler than expected, is the one most at risk of the Niedere Tauern lakes to future climate change^9^. It is likely that the lake will be subjected to marked reductions in the summertime catchment snow-cover and pronounced increase in the open-water season length^9^,^10^. The cold summertime epilimnetic water temperatures, which hardly exceed 10°C^11^,^12^, make Moaralmsee less stratified than lakes with warmer summertime temperatures, such as Twenger Almsee. Contrary to Moaralmsee, Twenger Almsee and Oberer Landschitzsee receive their water input mostly through precipitation and direct surface runoff, while the share of groundwater input is relatively small^13^,^14^. Oberer Landschitzsee was thermally stratified between ~1850 and 1950 AD, but it has apparently become a polymictic basin during the past decades^14^. In general, the water discharge, ice-cover duration and water temperature changes in the Niedere Tauern catchments in the 21^st^ century will likely far exceed the variations experienced at any stage during the last 10,000 years^9^. Furthermore, high altitude lakes in the Central Alps are likely to show maximum responses to climate change, because a one centigrade mean annual temperature change causes a 30 day change in ice-cover duration^9^. Consequently, climate warming can be anticipated to influence especially Alpine ecosystems situated on ecological boundaries^15^, such as the present study lakes, which are located in an ecotonal zone just above the tree line.

In contrast to the commonly used approach of using fossil Chironomidae (Insecta: Diptera) assemblages in lake sediments to reconstruct summer air temperatures, we here apply a novel approach of using a Chironomidae-based intralake calibration set to reconstruct past changes in summer water temperatures in the three study lakes. Fossil Chironomidae assemblages in surface sediments along a water depth/temperature gradient in Moaralmsee^11^ are used for the first time to develop a transfer function between the faunal assemblages and water temperature. Fossil Chironomidae are a well-established proxy for paleotemperature reconstructions^16^ and their within-lake water depth optima are strongly related to water temperature profiles^11^,^17^. Instead of the intralake multisample approach used in this study, Chironomidae-based temperature calibration sets are traditionally constructed from single-sample multilake data sets that tend to reflect the influence of regional summer air temperature rather than local water temperature^18^. Though water temperature directly influences Chironomidae development, growth, and survival, the relationship between Chironomidae distribution and temperature has been shown to be strongest in relatively deep, thermally stratified lakes where the macrobenthic communities live largely decoupled from the direct influence of air and surface water temperature^19^. This suggests that indirect effects of temperature on physical and chemical characteristics of lakes may also play an important role in determining the distribution of larval Chironomidae.

## Results

The Chironomidae-based water temperature inference model having highest coefficient of determination and lowest prediction error is developed with the weighted averaging (WA) technique when taxa tolerances are downweighted and classical deshrinking regression is used (Fig. 2). This model has a cross-validated (jackknifing) coefficient of determination of 0.81 and a root mean squared error of prediction (RMSEP) of 0.59°C with an average bias of 0.23°C and maximum bias of 1.37°C indicating that the model has good performance potential. The detrended correspondence analysis (DCA) scores (beta diversity) of Chironomidae assemblages have highest values in the initial part of all the sediment cores (Fig. 3). The highest variation in the DCA sample scores is in the Twenger Almsee core (2.2 SD) suggesting major faunal turnover, while the variations in Oberer Landschitzsee (1.3 SD) and Moaralmsee (1.3 SD) are lower, which indicate more subtle community turnovers. In Twenger Almsee and Oberer Landschitzsee the changes in beta diversity are concurrent with observed local temperature trends from Bad Ischl and the lowest values are found from the most recent samples. However, in Moaralmsee there is no relationship between air temperature trends and the DCA scores. The results of the reconstruction significance test (randomTF) show that the water temperature reconstructions from Moaralmsee (*p* = 0.04) and Twenger Almsee (*p* = 0.01) are statistically significant, whereas the reconstruction from Oberer Landschitzsee (*p* = 0.13) fail the test. The average observed July water temperature of 7.2°C, measured above the sampling site, is close to the reconstructed water temperature of 6.6°C in Moaralmsee. In Twenger Almsee, the average measured temperature above the sampling depth is 7.9°C, which is also within the model's prediction error (0.6°C), as the reconstructed value is 7.5°C. In both of these reconstructions, the sample-specific errors for the surface samples are 0.7°C, hence, the observed values are also within these error estimates. In Oberer Landschitzsee, the underestimation (1.0°C) of the reconstructed water temperature for the surface sample (8.7°C) is slightly over the model's prediction error and the sample-specific error estimate (0.6°C). However, the instrumentally measured values are single year measurements (seasonal and annual mean series), and therefore not directly comparable with the inferred value for the surface sediment samples, which corresponds to several years of sediment accumulation. The water temperature reconstructions from Twenger Almsee and Oberer Landschitzsee correspond with the observed air temperature increase, but in Moaralmsee the water temperatures show no increase as the inferred water temperatures vary within ~0.5°C throughout the core (Fig. 3). The increase in water temperature progressively continues in Lake Twenger Almsee until the present, but there is no increase in Oberer Landschitzsee since ~1950 AD. The increase in the inferred water temperatures from ~1800 AD to the present is ~4°C in Twenger Almsee and 2.2°C in Oberer Landschitzsee. The water temperature reconstruction from Twenger Almsee correlates strongly and statistically significantly with the instrumental summer (*r* = 0.90, *r*^2^ = 0.83, *p* = 0.001) and annual (*r* = 0.93, *r*^2^ = 0.87, *p* = 0.001) air temperatures. In Oberer Landschitzsee, there was no statistically significant correlation (*p*>0.05) between the inferred water temperature and observed summer (*r* = 0.23, *r*^2^ = 0.05) and annual (*r* = 0.45 *r*^2^ = 0.20) air temperature. Despite the very subtle changes in the inferred water temperatures in Moaralmsee, it shows significant correlation between the instrumental data during summers (*r* = −0.77, *r*^2^ = 0.53, p = 0.001) and also annually (*r* = −0.66, *r*^2^ = 0.37, *p* = 0.038). However, in contrast to the other lakes, the correlations in Moaralmsee were negative. Furthermore, in common with the *r*^2^ values, the *F*-test results for Moaralmsee indicate that the regression was not particularly powerful or significant at the level of *p* ≤ 0.01 (Table 1).

## Discussion

**Figure 1:**
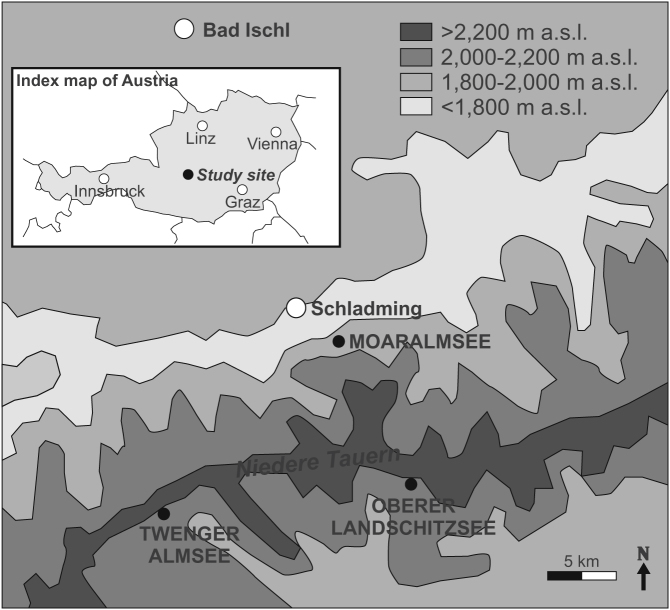
Study site. Location of the study site in Austria (index map) and the study lakes Twenger Almsee, Oberer Landschitzsee, and Moaralmsee in the Niedere Tauern Alps. Since Moaralmsee is located in the northern slope of the mountains, it is subjected to less insolation than the other lakes. The map is created with CorelDRAW version 15, Corel Corporation.

**Figure 2:**
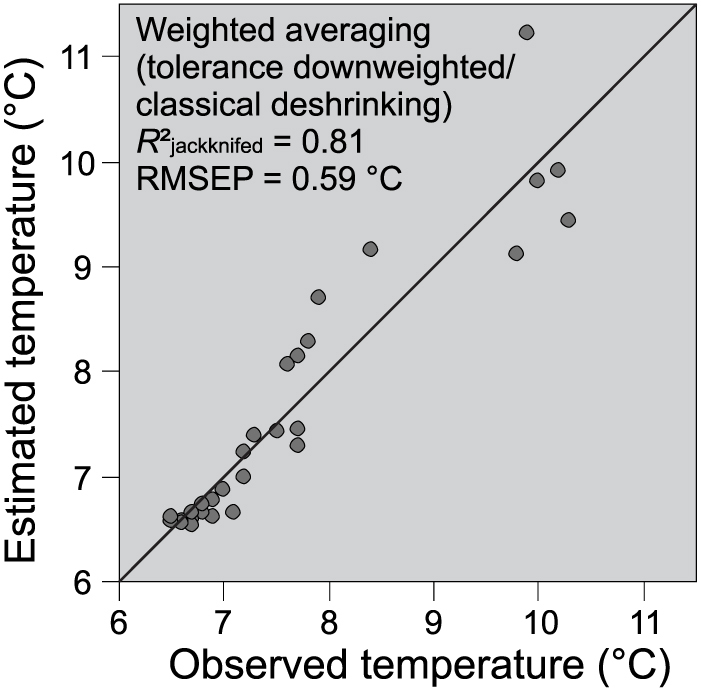
Relationship between measured and reconstructed water temperature. Chironomidae-inferred water temperature against instrumentally measured July water temperature from an intralake (Moaralmsee) calibration set.

**Figure 3:**
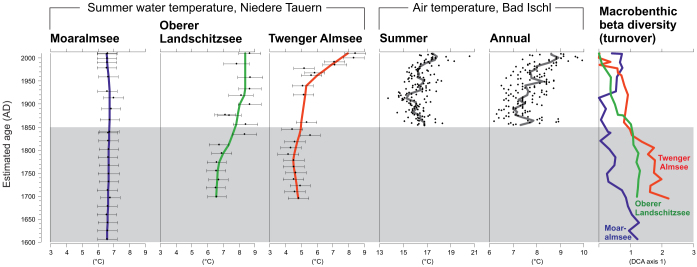
Summertime water and air temperatures in the Austrian Alps. Chironomidae-inferred July water temperatures in lakes Moaralmsee, Oberer Landschitzsee, and Twenger Almsee since the Little Ice Age (grey band) compared with instrumentally measured summer (June to August) and annual near surface air temperatures (single years smoothed with a 20 years low-pass filter) from an adjacent weather station in Bad Ischl (see location in Fig. 1) together with macrobenthic (Chironomidae) beta diversity (measured as detrended correspondence analysis axis 1 scores) indicating the rate of faunal turnover.

In opposite to the trend of recent climate warming, the water temperature reconstruction for Moaralmsee show no marked change during the past centuries, while the reconstruction for Oberer Landschitzsee and Twenger Almsee indicate increased water temperatures concurrent with the local air temperature increase towards the present (Fig. 3). However, there is no increase in the reconstructed water temperatures in Oberer Landschitzsee during the last century, which is likely caused by changes in summer stratification pattern as the lake apparently became polymictic after 1950 AD^14^ when the air temperature increase accelerated (Fig. 3). This is probably the reason why the inferred water temperature had no statistically significant correlation with the instrumental air temperature record. The strong correlation between water and air temperatures in Twenger Almsee suggests that the water temperature may have been driven by the increased air temperature in that particular basin. However, since both time series (water and air temperature) exhibit a positive trend, it could be the reason for the strong correlation by itself, and therefore does not provide evidence on causality. Another factor that hampers the reliability of the correlations is that they are based on a low number of samples (9–10). Nonetheless, the opposite negative correlation found in Moaralmsee suggests that there was no direct influence of climate warming on the water temperatures. Rather an indirect influence of climate change, possibly related to increased input of cold water from the melting snow patches and subsequent cold ground water, was driving the limnology of Moaralmsee. Furthermore, a cold microclimate caused by the shaded catchment on the northern slope of the Niedere Tauern Alps (Fig. 1) may have been the ultimate factor controlling the thermal conditions in Moaralmsee. However, it should be noted that although there was a statistically significant negative relationship between the reconstructed water temperature in Moaralmsee and the observed air temperature, the *F*-test suggests that the linear regression is not statistically significant when examined under the significance level of *p* ≤ 0.01 (Table 1). This is most likely due to the very subtle water temperature change in the Moaralmsee reconstruction.

The results also show that the ecological impacts of climate change are most subtle in Moaralmsee, as suggested by the trends in macrobenthic beta diversity (DCA axis 1 scores) (Fig. 3). In Oberer Landschitzsee and Twenger Almsee, the benthic beta diversity show contemporaneous trends with the instrumentally measured local temperature increase. However, there seems to be no correlation between the changes in beta diversity and reconstructed water temperature in Moaralmsee, suggesting that the macrobenthic communities were influenced by indirect climate impacts or by other environmental stressors such as intensified Alpine pasturing, atmospheric pollution, or changes in food web structure. In addition, lake level changes are a potential forcing factor for faunal changes in Moaralmsee, since it is a small basin without an inlet^11^.

Contrary to the general expectation that limnoecological conditions in lakes generally decrease under climate warming^20^,^21^, there are indications that invertebrate communities in Twenger Almsee have experienced a succession towards a state of increased oxygen availability^13^. Apparently, there has been a change in the lake's thermal structure, as summer mixing depth has deepened, causing a crossing of an ecological threshold. The change in the thermal structure of Twenger Almsee has likely been driven by the increasing air temperatures that have caused warming of the epilimnion and consequently, in combination with wind-induced mixing, deepening of the depth of summer thermocline. The deepening of the mixing layer has likely improved oxygen availability in the lake and subsequently improved the limnoecological conditions. Thus, the previous evidence on epilimnetic warming in Twenger Almsee are in agreement with the present water temperature reconstruction (Fig. 3). There has also been a change in the summertime mixing in Oberer Landschitzsee, since after the LIA the lake was first stratified causing oxygen deficiency, but then became polymictic during the 20^th^ century^14^. The current mixing pattern may contribute in explaining why the water temperatures have not continued to increase during the last decades.

The significance tests for the paleoenvironmental reconstructions show that the reconstructions from Moaralmsee and Twenger Almsee are statistically valid, whereas the reconstruction from Oberer Landschitzsee is not. The reason why the reconstruction from Oberer Landschitzsee was not statistically significant may be due to the linear temporal response of Chironomidae to environmental changes, as expressed by the DCA scores (Fig. 3). Hence, although the reconstruction significance test failed, the reconstruction may still be realistic. The reconstructed trends are in correspondence with the measured air temperatures, excluding the most recent decades when the relationship between air and water temperature broke due to the change in summer mixing pattern. Therefore, we interpret that the current reconstructions are realistic and reliable, but also note that the model used is probably most reliable in the focal lake (Moaralmsee), especially as the cores from Oberer Landschitzsee and Twenger Almsee were taken deeper than the training set maximum depth. However, reliable invertebrate-based paleoenvironmental reconstructions can also be achieved by using intralake models in non-focal lakes^22^. Most importantly, the modern thermal conditions of the lakes^9^ are suitable for the present model in all the sites.

Based on the present results and previous investigations^9^,^10^,^11^,^12^,^13^,^14^, it appears that watershed characteristics and lake stratification play a major role in defining the basin-specific thermal regime. In particular, the inferred temperature record at Moaralmsee indicates the importance of prolonged cold water inputs, such as snowmelt and groundwater. We also interpret that unlike in Twenger Almsee, water temperatures in Oberer Landschitzsee have not continued to increase during the recent decades due to its recent shifts in summer stratification patterns. Therefore, it is apparent that regional climate does not have the sole control on the lakes' thermal regimes. This study demonstrates that thermal development in lakes with similar prevailing climate conditions but different catchment and morphometric characteristics may have differing trends. Therefore, it is important to take into account catchment characteristics when discussing paleoclimate as interpreted through lake sediment cores.

## Methods

### Sites and sediment data

Lakes Twenger Almsee (47°13′N, 13°36′E; 2,118 m a.s.l.), Oberer Landschitzsee (47°14′N, 13°51′E; 2,076 m a.s.l.), and Moaralmsee (47°21′N, 13°47′E; 1,825 m a.s.l.) are cirque (corrie) lakes located in the Niedere Tauern Alps (Schladming) in Austria within ~20 km from each other (Fig. 1). The study lakes were chosen based on the previous assessment that they are particularly sensitive to climate change^9^. Twenger Almsee and Oberer Landschitzsee are located in the southern slope of the Niedere Tauern, whereas Moaralmsee is in the northern slope, and therefore receives less sunlight compared to the other study sites. The southern summits mainly dictate the insolation exposure for each of the lakes, and subsequently regulate the rate of summertime snowmelt. Due to its shaded catchment, Moaralmsee has persistent summertime snow patches in its catchment, as observed visually. The annual precipitation in the area is ~1800 mm, of which ~600 mm falls as rain during summer and the rest mostly as snow during autumn, winter, and spring.

Twenger Almsee is a deep (33.6 m) thermally stratified lake with a resilient thermocline, whereas Oberer Landschitzsee is a moderately deep (13.6 m) polymictic and Moaralmsee is shallow (7 m) polymictic lake. Despite their higher elevation, the average summer epilimnetic water temperatures in Twenger Almsee (~10°C) and Oberer Landschitzsee (10.5°C) are warmer than in Moaralmsee (~7.5°C)^6^. All the lakes are ultraoligotrophic and have small single outlets, but no inlets. More details about the study sites can be found from several previous publications^9^,^10^,^11^,^12^,^13^,^14^,^22^,^23^,^24^. Sediment cores from Twenger Almsee (22-cm), Oberer Landschitzsee (17-cm), and Moaralmsee (25-cm) were sampled in July-August 2010 from water depths of 18, 10.5, and 6 m, respectively, using a Kajak gravity corer to analyse fossil invertebrate remains at 1-cm intervals for assessments of long-term ecological responses to climate change^10^,^13^,^14^. The sediment record from Moaralmsee covers approximately the past 400 years^10^ and the records from Twenger Almsee and Oberer Landschitzsee the past 300 years^13^,^14^.

### Species turnover, proxy calibration, and water temperature reconstructions

Detrended correspondence analysis (DCA)^25^ is used to determine Chironomidae (macrobenthos) beta diversity in the sediment cores by applying the primary ordination axis scores (SD units). Subsequently, beta diversity is used to indicate the rate of faunal turnover in time. Intralake water temperature calibration set of Chironomidae assemblages in 30 surface sediment samples along the depth gradient (temperature profile) in Moaralmsee is developed using the weighted averaging technique. The observed water temperatures represent July measurements and the reconstructed temperatures characterize the conditions above the sampling site. To depict air temperature variability during the time covered by the sediment cores, we utilize the instrumental seasonal and annual mean series (available since 1860 AD) from a closely located weather station in Bad Ischl (47°71′N, 13°65′E, 512 m a.s.l.) (Fig. 1). Pearson product-moment correlation coefficient (*r*) and the level of statistical significance (significance levels of *p* ≤ 0.05 and *p* ≤ 0.01) are used to test the relationships between the water temperature reconstructions and observed air temperature data (20 year averages fitted individually to each sediment sample). The significance of the linear regressions is tested with coefficient of determination (*r*^2^) and the *F*-test. The statistical significance of the water temperature reconstructions are tested using the randomTF method^26^. This method is used to determine whether the present reconstructions explain larger proportion of the variance in fossil data than most of 999 reconstructions of random environmental data. Statistical significance test calculations are performed using the R statistical software^27^ package palaeoSig. The sample-specific errors in the reconstructions are estimated using bootstrapping cross-validation with 999 iterations.

## Author Contributions

T.P.L. designed the study. Field sampling and measurements were done by T.P.L. and L.N. and T.P.L. carried out the analyses. Both authors contributed to discussion, interpretation and writing the paper.

## Figures and Tables

**Table 1:** Pearson correlation (*r*), coefficient of determination (*r*^2^), *F*-test results, and the level of statistical significance (*p*) between the chironomid-inferred water temperature at the study sites and the measured air temperature from Bad Ischl

Site	Season	*r*	*r*^2^	*p*	*F*	*F* significance
Moaralmsee	Summer	−0.77	0.53	<0.01	11.5	0.01
	Annual	−0.66	0.37	<0.01	6.2	0.04
Oberer Landschitzsee	Summer	0.23	0.05	>0.05	0.4	>0.05
	Annual	0.45	0.20	>0.05	1.74	>0.05
Twenger Almsee	Summer	0.90	0.83	<0.01	28.2	<0.01
	Annual	0.93	0.87	<0.01	48.45	<0.01
